# The Test Pyramid 2.0: AI-assisted testing across the pyramid

**DOI:** 10.3389/frai.2025.1695965

**Published:** 2025-12-03

**Authors:** Priyank Desai, Snahil Singh, Shubham Amilkanthwar

**Affiliations:** 1 Independent Researcher, Seattle, WA, United States; 2 Independent Researcher, San Francisco, CA, United States

**Keywords:** software testing, quality assurance, artificial intelligence, DevSecOps, shift left, continuous integration, security testing

## Abstract

Ensuring robust test coverage, high code quality, and a strong security posture are persistent challenges in modern industrial software development, especially as systems grow in complexity and release cycles accelerate with recent Artificial Intelligence (AI) related productivity gains. This paper introduces a conceptual framework, "The Test Pyramid 2.0", which offers a clear and actionable path to integrate the latest advances in AI and DevSecOps principles into engineering workflows to achieve greater efficiency, reduce defect leakage, and create more resilient systems. We examine how AI enhances each layer of the test pyramid through capabilities such as automated test generation, coverage analysis, test data synthesis, anomaly detection, and intelligent UI exploration. In parallel, we embed DevSecOps practices directly into the pyramid by aligning security controls with each testing layer, ranging from static analysis and policy enforcement to dynamic testing, misconfiguration detection, and adversarial simulation. We also explore how AI strengthens these security practices through adaptive learning, risk prioritization, and context-aware detection. Together, these advances create a holistic, AI-augmented, and security-conscious testing strategy that supports the speed of modern development without compromising quality or safety.

## 1 Introduction and background

Testing remains a cornerstone of modern software delivery. Existing challenges in ensuring robust test coverage and maintaining a strong security posture are further exacerbated by unprecedented productivity gains achieved through the use of Generative AI in the software development life-cycle (Rolls, 2025). As developer velocity increases, so does the urgency to ensure that production-ready software is backed by automation-enabled quality and security practices. In addition, for practitioners, test coverage alone is not sufficient; automated testing and fast feedback loops are essential to support rapid iteration without compromising reliability or safety (Shahin et al., 2017). Meanwhile, according to the DevSecOps philosophy, Security must also “shift left,” becoming a first-class citizen in testing strategies rather than an afterthought of deployment (Lietz, 2015). This paper addresses these needs by re-examining the classical test pyramid through the dual lenses of AI and DevSecOps.

**Figure 1:**
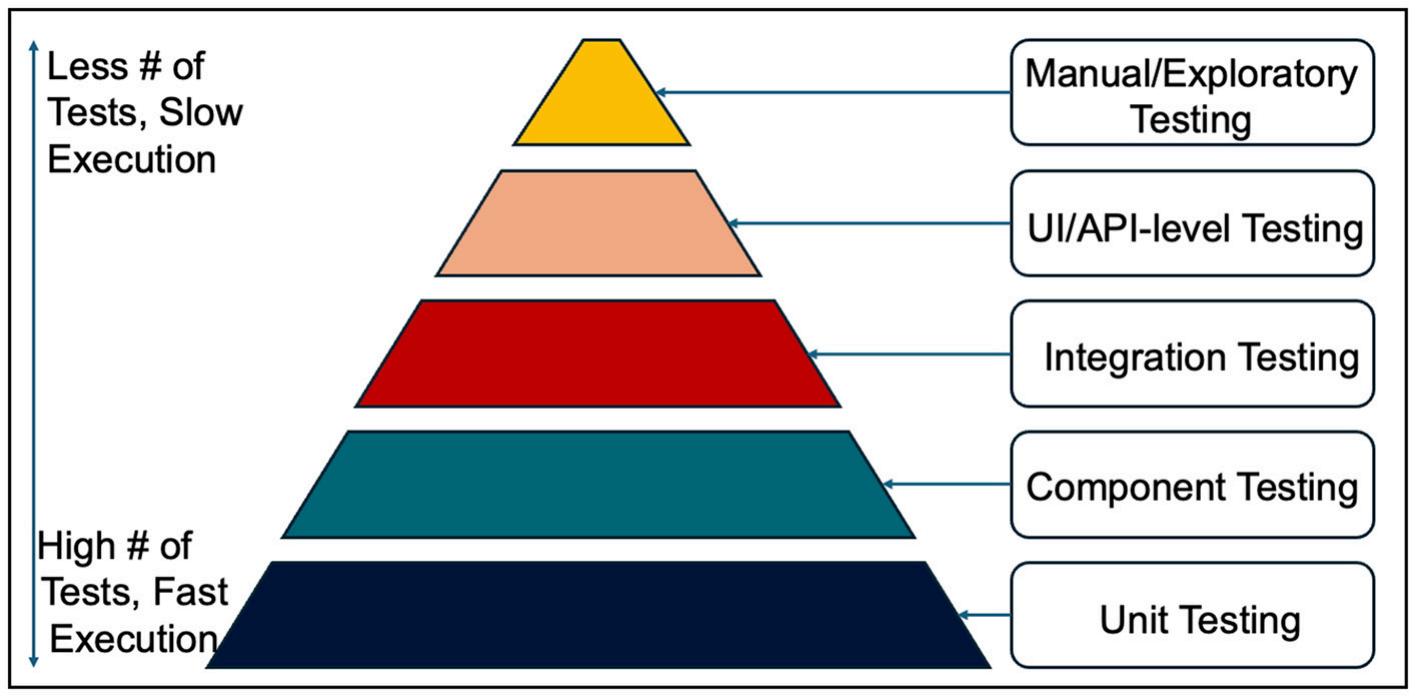
The Test Pyramid.

“The Test Pyramid” (Cohn, 2009) is a fundamental and widely adopted concept in modern software quality engineering. From unit, component and integration tests to UI/API and manual exploratory tests, it is a structured framework for balancing effort, isolation, and speed in different layers of application testing. Originally proposed by Mike Cohn and expanded by various practitioners such as Vocke (2018), the pyramid encourages teams to build a test suite that is comprehensive, efficient, and maintainable. The basic principle is that tests become broader, fewer, and more expensive to execute as we move up the pyramid. This structure emphasizes speed and feedback efficiency at the lower levels, while it captures essential behavioral checks at the upper layers (Figure 1). The concept has been widely adopted in Agile environments and forms a cornerstone of scalable test automation strategies.

We extend this model into “The Test Pyramid 2.0,” embedding security testing directly into each layer. It is important to note that in this paper we operate on a more granular version of the Test Pyramid proposed by Mike Cohen in his book “Succeeding with Agile: Software Development Using Scrum.” The original test pyramid had three layers (Unit, Service, and UI/E2E), while we operate on a pyramid with five layers that incorporate various testing methodologies used across the industry and academia today. This allows us to dive deeper into the advances and vend our recommendation at each granular layer.

Further, we explore the role of AI in amplifying both quality and security outcomes. From automated test generation, intelligent test orchestration, and anomaly detection to adaptive vulnerability scanning and context-aware risk prioritization, various AI techniques transform each layer of the pyramid into a smarter, more proactive quality and security checkpoint. By framing these advances within a familiar model, this document offers a practical framework for teams seeking to deliver faster, safer, and more resilient software in today's high-velocity engineering environments.

## 2 Changes to the test pyramid—The Test Pyramid 2.0

**Figure 2:**
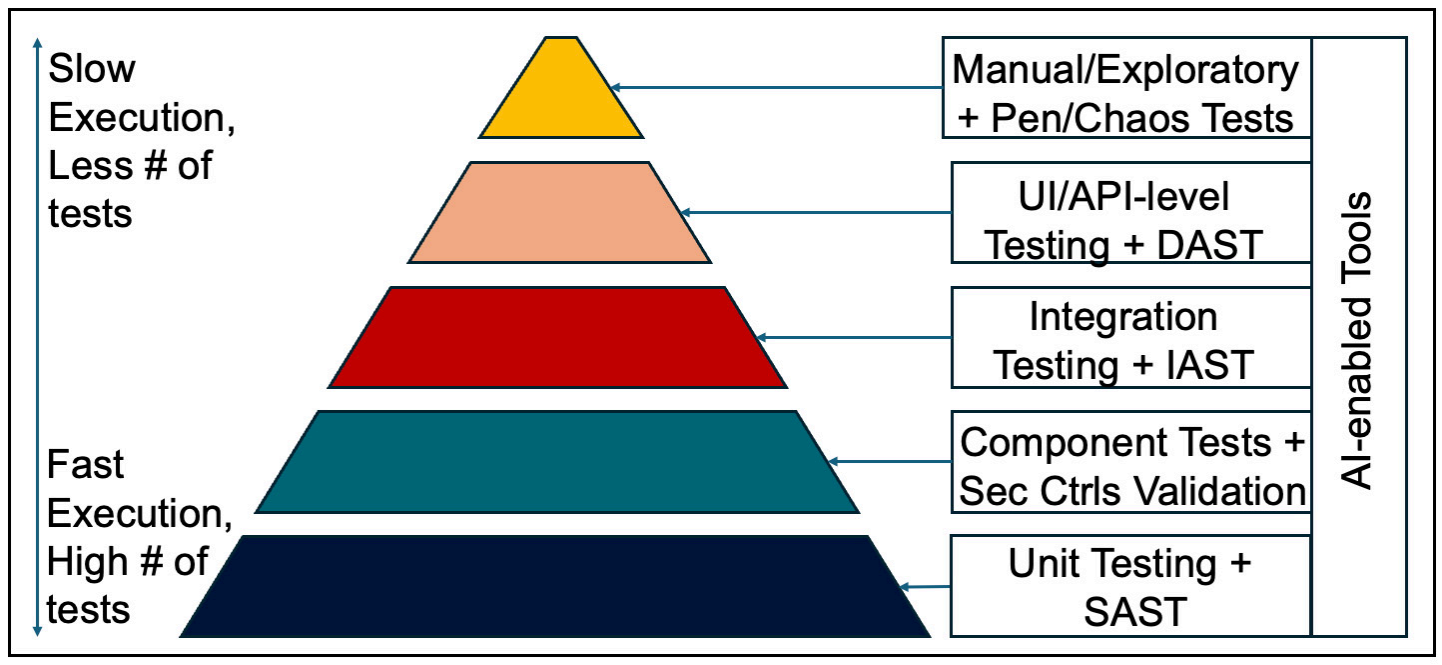
The Test Pyramid 2.0.

In each sub-section below, we start by defining the traditional role of each layer in the test pyramid. We then cover the proposed changes: (a) application of various AI techniques based on existing research and (b) embed a concrete suite of tests borrowed from the DevSecOps philosophy. These changes are summarized in Figure 2.

### 2.1 Unit testing (Layer-1)

Unit tests form the foundation of the pyramid and are the most comprehensive in terms of code coverage. These tests focus on the narrowest scope, typically a single function or method at the package or module level. The goal is to validate that individual units of code behave as expected for various inputs, often through stubbing or mocking external dependencies to ensure isolation. Due to their lightweight nature and fast execution time, unit tests provide the quickest feedback loop and are critical for supporting rapid development cycles. Obtaining close to 100% coverage at this level is generally considered ideal. Their close relationship with code modules makes them prime candidates for AI-driven automation. Here are some of the automation techniques that we recommend applying at this layer:

#### 2.1.1 Automated testcase generation

A broad spectrum of studies have been published that used Large-Language Models (LLMs) to generate test-cases. Chen et al. (2024) proposes “ChatUniTest,” which is a framework for LLM-based Test Generation. The authors use four Java projects (from different domains eCommerce, Binance connectors, etc.) and compare it's performance with “EvoSuite” (Fraser and Arcuri, 2011) which is based on evolutionary algorithms. Compared to the overall coverage of 38.2% achieved by EvoSuite, the LLM-based solution yielded 59.6%. Further, Adu (2024) discusses Prompt engineering, Fine-tuning, and Retrieval-Augmented Generation (RAG) techniques. Through experiments, the research shows the performance improvement of an LLM (gpt-3.5-turbo), generating up to 85% relevant tests, with the remaining 15% scenarios deemed Minor (not important; good to have).

In addition, studies such as Takerngsaksiri et al. (2025), provide a solution to generate automated test-cases using Deep Reinforcement Learning. This text-to-testcase generation approach enables test-driven development (TDD), where code is not available for testcase generation. Finally, Baqar and Khanda (2025) discusses case studies that demonstrate how AI can effectively enhance testing efficiency in both legacy and modern software systems. Building on these prior researches, we see LLMs (along with other AI techniques like RL playing a smaller role) as a potent tool to generate unit test cases by parsing code structures, identifying logical branches, and proposing scaffolds that test key execution paths, providing a valuable tail-wind for users in both industry and academia.

#### 2.1.2 Code coverage analysis

As mentioned in the above section, LLMs can help automate test case generation, which improves code coverage. However, the application of Reinforcement Learning (RL) can further improve code coverage. In CGFT Engineering (2025), the authors used test-coverage guided RL to fine-tune a model with 7B params and deployed a custom unit-testing agent. The agent significantly improved code coverage (at a coverage increase rate of 0.41), outperforming general-purpose models like o3-mini/o4 (at a 0.3 increase rate), even while being relatively small @ 7B params. Similarly, in Zhang et al. (2025), researchers optimized LLMs for unit test generation via chain-of-thought (CoT) prompt and reinforcement learning (RL) from coverage feedback. This strategy helped LLMs understand the semantic intricacies and logical constructs, along with the diversity of the generated tests. Such AI-enhanced tooling can provide real-time code coverage insights, identifying untested paths, and auto-generating tests to fill those gaps. Smith (2024) offers a sample methodology along with some case studies.

Furthermore, we envision that such tools will increasingly be integrated with code editors to offer live assessments of cyclomatic complexity, refactor suggestions, and maintainability scores, helping elevate code quality along with test completeness. Almeida et al. (2024), presents a research investigation into the application of Artificial Intelligence to enhance the quality and efficiency of code. An IntelliJ IDEA plugin was developed to achieve this objective, leveraging GPT-3.5 as the foundational framework for automated code assessment. The researchers tested the tool in a test group and control group setting and found: (a) a higher average of 28 code smells compared to the control group's 20 and (b) detection of 25 refactored code smells on average, as opposed to the control group's 13. By automating test generation and providing intelligent feedback during development, we visualize AI enabling teams to scale their unit testing practices more effectively and with greater precision.

#### 2.1.3 Introducing static application security testing in Layer-1

In the updated test pyramid, we extend the base layer to include Static Application Security Testing (SAST) alongside traditional unit tests. SAST analyzes the source code to identify common security vulnerabilities such as injection risks, hard-coded secrets, or unsafe API usage, well before the code reaches production. Embedding SAST at this foundational level aligns with shift-left principles and ensures that security becomes a default part of the developer workflow, not a delayed checkpoint. SAST's frequency, proximity to the source code, and fast execution speeds are our primary motivations for aligning it with the unit testing layer.

Artificial Intelligence meaningfully enhances the effectiveness of this layer. Using a large corpus of known vulnerabilities and secure coding patterns to learn, Russell et al. (2018) demonstrated the potential of using ML to detect software vulnerabilities directly from the source code. The study experiments with a variety of ML techniques and achieves the best overall results using features learned through a convolutional neural network (CNN) and classified with an ensemble tree algorithm. Such AI-augmented SAST tools can detect complex or context-dependent issues that traditional rule-based scanners often miss. In addition, Santos et al. (2021) presents a comparison of manual code review, traditional SAST tools, and SAST tools with ML to provide a starting point for organizations. Involvement of ML also ensures that these systems continuously adapt to emerging threats, evolving their detection capabilities in response to new Common Vulnerabilities and Exposures (CVEs), code-bases, and language ecosystems.

One of the most impactful contributions of AI here is the reduction of false positives, a long-standing challenge in static analysis. Klieber and Flynn (2024) uses LLMs, which show promising initial results in adjudicating static analysis alerts and providing rationales for adjudication, offering possibilities for better vulnerability detection and reducing false positives. Through a deeper contextual understanding of code flow and function interactions, the researchers aim to enable LLMs to improve the signal-to-noise ratio and developer trust in these tools.

Combining unit testing with intelligent static analysis at the base of the pyramid allows teams to catch functional and security defects early, resulting in faster remediation, better security posture, and stronger development velocity.

### 2.2 Component testing (Layer-2)

Located above unit tests in the pyramid, component tests validate the interaction between a small number of units or methods. They are designed to test logical groupings of code, methods, or functions, often by mocking remote endpoints, aiming to verify internal behaviors and relationships. These tests still maintain relatively fast execution times and contribute significantly to understanding behavioral patterns within components. Although slightly less granular than unit tests, they provide broader insight into how different units collaborate.

#### 2.2.1 Automated testcase generation and code coverage

Like unit testing, component tests are numerous and fast, and their proximity to business logic makes them ideal for high-coverage functional testing using the AI-powered automation mentioned above in Sections 2.1.1, 2.1.2. For automated test case generation and analyzing/improving code coverage, we visualize AI to assist this layer using the same techniques as Layer-1. The goal is for teams to continue to make it easier to validate component-level behavior independently of the larger system context.

#### 2.2.2 Smart test maintenance and self-updates

As component tests often span functions, methods, and modules, even small changes to the code-base can break numerous tests. Advanced tools have historically provided features for updating tests in response to changes in source code; however, applications of AI like Code Language Models (CLMs) significantly improve this capability. Saboor Yaraghi et al. (2025) introduces TaRGET (Test Repair GEneraTor), a new method that utilizes CLMs to automatically repair test cases. The best performing model in this rigorous study achieves 66.1% exact match accuracy (EM) and 80% plausible repair accuracy (PR) on an extensive benchmark. Recent advances in the field have further improved the capability of CLMs, giving way for Large Language Models for Code Understanding and Generation. In Wang et al. (2023), researchers explore a new family of open code LLMs to support a wide range of tasks. In this study, the researchers verified the code-LLMs via extensive experiments on more than 20 code intelligence benchmarks.

Additionally, recent studies such as Pathik and Sharma (2022) focus on the use of deep learning models to perform an accurate impact analysis of code changes. With a clear understanding of the semantic relationships between different elements of the code, teams can create automation that can reliably predict the ripple effects of a change and update tests automatically. Researchers achieve a precision of up to 97.2% using their proposed approach. Given all these advances, we recommend that industry and academia teams take advantage of such AI-enabled tools to detect outdated or broken tests and proactively update them before execution, saving significant engineering time and bandwidth. This is particularly valuable in complex applications with large, interdependent components, where maintaining test suites manually becomes increasingly burdensome.

#### 2.2.3 Enhanced test execution and orchestration

Two primary issues for test-driven development (TDD) and continuous integration (CI) are rapidly changing environments and slow test execution, both leading to slower developer feedback. AI is poised to make meaningful improvements to both these issues. Bagherzadeh et al. (2022) highlights an RL-based approach for Test Case Prioritization, with prioritization strategies that produce high accuracy [Normalized Rank Percentile Average (NRPA) > 0.96], approaching the optimal ranking of the test cases based on actual failure data and execution times. Similarly, Agentic Orchestration platforms like Mathew (2025) optimize test execution, balance test loads, and predict flaky tests. Such capabilities accelerate the feedback loop, where individual developers benefit from faster results, and engineering teams gain greater velocity and confidence in the code-base's behavior at the component level.

#### 2.2.4 Introducing security controls validation in Layer-2

In the second layer of the updated test pyramid, we propose extending component testing to include Security Controls Validation (SCV), a DevSecOps practice focused on verifying that an application's security measures work as intended. In other words, SCV ensures that the security mechanisms themselves are functional, enforced, and resilient in production; it bridges the gap between secure development and secure operations. This involves systematically evaluating enforcement points, such as access control policies, data handling rules, and configuration settings within individual components. We see this layer as a natural place to codify and enforce security policies as code (PaC), given the cost of executing tests in the higher layers. Also, security controls validation does not need to rely on component interactions, aligning well with Layer-2 restrictions.

Recent advances in AI have made way for research such as Romeo et al. (2025) an agentic system that combines LLMs, Retrieved-Augmented-Generation (RAG) and tool-based validation to automate the generation and verification of PaC rules. Through experiments, researchers baseline different LLMs (Claude Sonnet 4, GPT-4o, and Qwen3:30b) in plain-LLM, RAG, and Agentic RAG setups and show that Claude Sonnet 4 is able to generate Rego rules without external knowledge and also verify them on the test application. Similarly, Nobi et al. (2022) surveys and summarizes the emerging benefits of machine learning in various areas of access control, including attribute engineering, policy mining, and access control policy verification. Policies could come from internal governance rules, compliance frameworks, or cloud configuration baselines. Solutions like this allow teams to catch policy non-compliance before code moves to higher layers of testing.

At this level, AI-techniques like ML and LLM have been shown to automate vulnerability scanning and identifying security misconfigurations. In addition, Shiri Harzevili et al. (2024) shares a systematic literature review on Automated Software Vulnerability using various Classic and Deep Learning ML models. While Wen et al. (2025) highlights the use of LLMs for misconfiguration detection, outperforming existing data-driven approaches with a precision of 72.88%, recall of 88.18% and F1-score of 79.75%. Teams can deploy such solutions to continuously analyze application components and infrastructure definitions for insecure configurations, drift, or known weaknesses.

Embedding security controls validation into the component testing layer ensures that security policies are not just present but effective, and that violations are caught early, while changes are still isolated and inexpensive to fix. This is key to our vision of making this layer a crucial checkpoint for building secure systems from the inside out.

### 2.3 Integration testing (Layer-3)

As we ascend the test pyramid, the emphasis shifts from code-level precision to validating cross-component interactions and system behaviors. Integration tests reside in the third layer of the pyramid and focus on system-level behaviors rather than isolated units. These tests are generally fewer in number compared to the previous layers' tests but are vital to verify that distinct services or modules communicate and function together as expected. Unlike unit or component tests, integration tests typically do not mock remote services and are often run in controlled environments such as staging or beta environments. As an example, a common use-case involves verifying database integrations to ensure that the entire stack behaves correctly with databases under real-world conditions.

Below, we explore how AI can augment this layer by addressing longstanding challenges related to test data fidelity and use intelligent defect targeting to further improve effectiveness.

#### 2.3.1 Testdata generation and management

A persistent challenge in integration testing is managing test data across environments. Unlike unit or component tests, integration scenarios typically involve communication between multiple subsystems. This often requires that test entities exist in a specific (and valid) state before testing begins. For example, a test verifying a user's subscription retrieval must ensure that the user account exists and has associated subscription history configured.

Existing research like Behjati et al. (2019) has proposed the use of Recurrent Neural Networks to generate synthetic test data, however advances in LLM technology have unlocked additional capabilities. In Baudry et al. (2024), researchers experiment with GPT-4 and show improved data adequacy (63 case studies), executability (69% success rate), and high-degree of compatibility with existing faking libraries. Such AI-powered tools reduce the manual effort required to provision and maintain contextual test data. Teams can leverage them in an attempt to generate (or clone) valid entity states and ensure data consistency across test runs. Long et al. (2024) surveys the current available research on LLM-driven test data generation and articulates directions for future research for adoption in diverse organizations. In addition, the enhanced ability to generate synthetic data helps teams ensure compliance with privacy and data protection standards.

In addition, studies such as Rashidi et al. (2024) show the role of ML in the quality of synthetic data generated for tests. Researchers embedded an auto-ML module in the loop, allowing users without deep expertise to generate usable synthetic datasets and fully automating the synthetic data generation.

#### 2.3.2 Defect prediction and risk analysis

ML techniques enable teams to intelligently forecast where defects are most likely to occur within or between integrated components. In Madeyski and Stradowski (2025), researchers propose a lightweight approach for ML-based Software Defect Prediction (SDP), along with the use of explainable AI to provide feedback to stakeholders. They used a real world scenario to validate their solution, vended the solution to professionals, and solicited feedback from industry experts to achieve high precision (up to 0.981). In practical scenarios, various features such as historical bug patterns, test reports, change frequency, and dependency graphs are available for ML models to identify and highlight risky areas. In addition to applications that use ML, several DL-based mechanisms have also been proposed that exploit such features (Giray et al., 2023). Insights from such solutions help engineering teams allocate testing resources strategically (to focus on defective areas) and preempt issues before they reach production.

Separately, the authors of “The use of artificial intelligence for automatic analysis and reporting of software defects” (Esposito et al., 2024) concluded that companies that incorporate AI algorithms will be able to include an agile model in their life cycle, as they will reduce the rate of failures, errors, and breakdowns, allowing cost savings and ensuring quality. When paired with automated test generation, we visualize defect prediction capability to target risk areas and produce high-value test cases, effectively closing the loop between prediction and prevention. We see this approach as not only improving test coverage but also helping teams avoid wasted cycles in low-risk areas.

#### 2.3.3 Introducing interactive application security testing in Layer-3

In this layer of the enhanced testing pyramid, we expand integration testing to include Interactive Application Security Testing (IAST), which is a dynamic technique that observes application behavior, data flow, and interactions during runtime. IAST uniquely provides accurate, runtime-verified vulnerability detection with full code-level context. Since integration tests validate how components work together at runtime, this is a natural point to embed IAST as it instruments the running application during test execution, offering real-time insights without requiring security scripts. This layer provides an opportunity where it is still early in the development cycle to allow for quick remediation, yet rich in execution context.

Researches like Wang et al. (2025) have proposed the use of LLMs to identify data flows across component boundaries. This is a critical capability as it allows IAST to dynamically detect data flows. Models can now learn and establish behavioral baselines for how components typically interact. When these patterns deviate, they become a signal of potential vulnerability; researchers in Chang et al. (2025) use CNNs with such signals for anomaly detection. Their algorithm algorithm achieves an average accuracy of 95.88% a recall rate of 91.23% and a false positive rate of 2.34%. Such solutions adds net value in addition to standard rule-based detection, which is widely used in existing IAST tools.

Recent research like Mehmood et al. (2023) and Basheer et al. (2024) also presents approaches that use ML to detect issues like privileged access escalation through chained components and insecure API invocations. The former achieved accuracy of up to 97% in detecting and classifying a Privilege Escalation Attack, while the latter achieved up to 88%Ṫhese are often difficult to detect with static rules alone, but become apparent when ML models learn the expected patterns and spot deviations.

Finally, similar to previous layers, AI-techniques can be leveraged to generate targeted test data as security testing often requires crafted inputs to probe for specific vulnerabilities such as injection vectors or malformed headers. Meng et al. (2024) employs the use of LLMs to mutate inputs for broad code coverage. Using this solution, the authors discovered 9 distinct and previously unknown zero-day vulnerabilities in widely-used and extensively-tested protocol implementations, while the baseline tools only discovered 3 or 4 of them.

By combining integration testing with AI-enhanced IAST, this layer becomes a powerful mechanism to uncover complex, cross-service security flaws early in the life cycle.

### 2.4 UI/API testing (Layer-4)

UI and API tests operate on the user interface or the external interface of the application. This is the fourth layer of the pyramid and generally comprises the final level of automated testing. These tests assess how the system behaves from an end-user or external consumer (client application) perspective, and hence play a critical role in ensuring end-to-end functionality. Depending on the application, this may involve graphical UIs, RESTful APIs, etc. These tests also verify the integration of the application with external systems and services. Given their broader scope and dependency on full system contexts, they have the slowest feedback cycle and the lowest isolation among automated tests. Given their broad scope and proximity to real-world usage patterns, this layer presents unique opportunities for various AI techniques to improve the quality, speed, and efficiency of tests, as described below.

#### 2.4.1 AI-enabled behavior driven development and testing

At this level, we conceptualize the use of Generative AI to create Behavior-Driven Development (BDD) (Terhorst-North, 2006) test-cases, along with the corresponding test code. Chemnitz et al. (2023) proposes a similar vision for the generation of source code from the BDD syntax; however, we propose deploying GenAI for the creation of tests and test code. By feeding AI systems with Business Requirements Documents (BRDs), which are standard in the software development industry, teams can generate BDD-aligned test cases that enable them to test the behavior of the target application.

BDD emphasizes the creation of tests based on expected user behavior and business requirements, often written in human-readable formats such as Gherkin. Meanwhile, Generative AI thrives on large and well-structured textual data (Moyer, 2025). We envision tools that leverage these capabilities and generate BDD tests (from BRDs) to reduce the developer effort and improve coverage. Reliance on text/specs also helps improve collaboration between product, development, and QA teams, ensuring clearer specifications and earlier detection of misalignment while producing and managing essential test cases. The result is software that better aligns with user expectations and business goals, supported by a comprehensive and human-readable acceptance testing framework.

#### 2.4.2 Visual testing

AI has expanded the scope of visual regression testing. Instead of relying on brittle pixel-by-pixel comparisons, AI-enabled tools use object recognition and contextual awareness to evaluate visual correctness. For example, Gamal et al. (2023), researchers propose Owl Eye, which is an AI-driven visual testing tool. It uses a combination of traditional image processing and deep learning to identify visual defects in GUI testing, achieving an F1-score of 72%Ṡimilarly, in Komar et al. (2024), researchers use neural networks to detect layout changes, misalignments, color inconsistencies or missing elements, many times in varying screen sizes and browsers. This form of intelligent visual validation supports consistent user experiences, particularly in modern multi-platform applications where UI variations are common. We expect development of capable AI agents can also maintain baseline images, highlight only meaningful differences, and ignore inconsequential changes like anti-aliasing or font rendering.

#### 2.4.3 Improved defect identification and reporting

We envision that AI tools (and agents) at this layer can go beyond simply executing tests. LLMs can be used to analyze logs, outputs, and API responses to detect anomalies, correlate error patterns, and categorize failures. Researchers in Anjali et al. (2023) employ ML techniques to automatically identify probable root causes and recommend next steps for classification and resolution, achieving an accuracy of 96.56%Ȧlso, similar to SDP applications, AI agents can learn from historical defects, error reports, and behavioral patterns to detect and report failures.

Researchers have also used LLMs to improve the quality of bug reporting. Acharya and Ginde (2025) demonstrates how instruction fine-tuned LLMs can automatically convert unstructured bug reports into well-structured ones that closely follow standard templates. In their study, the fine-tuned Qwen 2.5 achieved a CTQRS (Crowd-sourced Test Report Quality Score) of 77%. Such detailed reporting capabilities can provide actionable diagnostics to developers, while Quality Assurance engineers can gain visibility into recurring trends or system hot spots, enabling both the roles to effectively partner in expediting development cycles.

#### 2.4.4 Introducing dynamic application security testing in Layer-4

At the top of the automated testing stack, we extend traditional UI and API-level testing to include Dynamic Application Security Testing (DAST). Similarly to UI/API Testing, DAST is a black-box testing technique that simulates attacks against a running application to identify vulnerabilities that an external attacker could exploit. Unlike static analysis, DAST does not require access to source code as it evaluates the application as an outsider, making it a natural fit for this layer, where automated tests validate system functionality across real endpoints and user flows. As the highest layer with automated testing, this is the ideal point for simulating real-world security threats and evaluating how the system responds under attack.

One of the persistent challenges in DAST is the accuracy of crawling dynamic and complex applications to uncover all available endpoints and inputs. Recent research has shown significant promise in the discovery of applications' attack surface. For example, Yang et al. (2025) proposes “CrawlMLLM,” a framework using multi-modal large language models to simulate human web browsing. It attempts to address the challenges of complex page relationship discovery and contextually inappropriate input generation to identify attack surface. In six real-world applications, CrawlMLLM detected 20 vulnerabilities while the next best method found six. Similarly, Varga (2025) proposes an ML-enhanced web-crawler for vulnerability detection. Such solutions can substantially enhance DAST's effectiveness by identifying the full attack surface, including hidden routes, conditional flows, etc. and ensuring that testing is not limited to known paths.

Next, similar to Section 3.3.1, various AI techniques can be employed to generate synthetic data input that can simulate vulnerabilities such as SQL injection or cross-site scripting (XSS). Jha et al. (2024) proposes a BERT and Reinforcement Learning Based fuzzer that helps identify vulnerabilities. The RL-guided feedback loop enables it to automatically generate and search the space of attack vectors to exploit the weaknesses of the given victim application without the need to create labeled training data. The researchers observed a significant improvement in terms of time to first attack (54% less than the closest competing tool). By learning which vectors are the most successful against specific technologies or patterns, the solution improved both detection accuracy and testing efficiency over time.

By combining UI/API-level testing with AI-augmented DAST, this layer delivers high-value security insights and identifies issues before they reach production. It is important to understand that the addition of DAST at this layer makes it heavier and more expensive; we have provided some high-level insights later in the paper, while a deeper cost-benefit analysis will be covered in future work for this manuscript.

### 2.5 Manual or exploratory testing (Layer-5)

At the top of the pyramid lies manual or exploratory testing, sometimes referred to in industrial settings as "Friends and Family" testing or acceptance testing. This stage focuses on unscripted human-centered evaluation, where testers creatively explore a running system to identify quality issues, usability challenges, or unexpected behavior. These tests are valuable for surfacing real-world issues that are not captured by scripted tests. The ideal approach is to automate the insights gained from the exploratory sessions, thereby enriching the automated test suite over time. With the rise of generative AI and autonomous agents, even this layer is poised to see a meaningful transformation.

#### 2.5.1 Generative AI for scenario exploration

We expect teams to be able to use Generative AI (GenAI) tools to simulate various user interactions, exploring combinations of actions and edge cases that may not be explicitly covered in business requirements, coded assumptions, or test cases. These tools can act as “imaginative testers,” proposing scenarios that go beyond what the engineering team anticipated. Pyhäjärvi, (2025) narrates the perspectives of practitioners' in using AI as a tool for exploratory thinking. By identifying boundary conditions and behavioral inconsistencies, GenAI can enhance the depth of exploratory testing and reduce the likelihood of latent bugs escaping into production.

Although this layer has historically been manual, the emergence of AI agents is promising. As agents become more capable of automating manual human tasks, we envision that exploratory QA can be led by AI agents, enabling continuous, intelligent exploration that adapts as the system evolves.

#### 2.5.2 Introducing penetration and chaos testing in Layer-5

At the top of the testing pyramid, we broaden the traditional scope of manual and exploratory testing to include Penetration (Pen) Testing and Chaos Testing, both of which are essential for validating a system's resilience against real-world threats and failures. Penetration testing simulates the behavior of an attacker to uncover exploitable security vulnerabilities. Similarly, Chaos Testing intentionally injects failures into a system to evaluate its ability to withstand unexpected disruptions. Like exploratory testing, both of these are largely driven manually and are often reserved for critical checkpoints because of their cost and effort.

In penetration testing, we envision GenAI (LLMs) to help in various steps of the process, such as the planning phase, identifying potential attack paths, and prioritizing vulnerabilities. In Ćirković et al. (2025) researchers apply LLMs for the automated generation of malicious payloads in penetration testing processes. They successfully generated advanced payloads for three key types of attacks: XSS, SQL Injection, and Command Injection, where the total time required to generate a single synthetic attack was 10.5231 s. Their experimental results confirm that this approach not only reduces the time required for penetration test but also significantly improves the coverage and accuracy of vulnerability detection compared to traditional methods.

We envision similar benefits for chaos testing. Hernández-Serrato et al. (2020) visualizes machine learning techniques that can learn from existing a-priori data, stream data, or both to address various challenges in automating chaos testing. They propose ML applicability in data labeling, feature engineering, system modeling, experiment management, post-mortem analysis, and automated recovery, covering the full life-cycle of chaos tests. At this layer of the pyramid, the system is still under test and does not serve production traffic, allowing automation to iteratively cycle components offline and online and adapting fault patterns, without causing production customer impact.

Together, these practices elevate the top of the pyramid from occasional manual testing to strategic simulation and resilience validation, with different AI techniques acting as an intelligent orchestrator of deeper, broader, and more adaptive test coverage.

The diagram below summarizes our concept of the “The Test Pyramid 2.0” in it's entirety.

## 3 Cross-cutting improvements with AI

While the previous sections examined how various AI techniques can augment each layer of the test pyramid, this section explores the broader, cross-layer capabilities that AI unlocks and how it can unlock holistic improvements to testing strategies, pipelines, security, and overall software quality practices.

### 3.1 Use of generative AI to develop a comprehensive QA strategy

Beyond individual tools and workflows, we envision engineers and leaders to partner with Generative AI capabilities (of existing LLMs) to craft end-to-end quality assurance strategies. This involves selecting the right AI-driven tools and models for different layers of the pyramid, aligning them with organizational goals, and integrating them into a cohesive test capability. By incorporating critical productivity and efficiency metrics (aimed at performance evaluation, impact assessments, and observed quality/security improvements) as a feedback loop, teams can continuously refine their QA strategy. This “learn and adapt” loop enables organizations to drive consistent efficiency gains and increase the resilience of their software systems over time.

### 3.2 Agentic testing across the pyramid

The emergence of Autonomous AI agents capable of navigating software applications autonomously opens new possibilities. Although research in this area is still nascent, industrial applications of AI agents are on the rise. Andrades (2025) discusses the use of AI Agents to autonomously drive self-healing testing scripts, self-learning and adaptive tests, predictive error detection, continuous optimization, and autonomous test implementation. Kumar (2025) covers industrial case studies on the use of AI Agents in testing, where an online retailer (eCommerce application) reported a 95% reduction in the maintenance effort of their tests.

Although this is still an emerging area, agentic testing represents a promising avenue for future investment. In the longer term, we envision the entire test pyramid, including the traditionally manual top layer, to be automated through intelligent agents that cover testing for application and security defects.

## 4 Discussion and practical considerations

AI-augmented testing combined with embedded DevSecOps principles holds significant promise, but realizing its full potential requires intentional strategy and cultural adaptation. The goal is not just to automate more, but to integrate quality and security thinking into every stage of delivery and turn AI into a trusted partner rather than a disconnected tool.

### 4.1 Role evolution for developers and quality assurance engineers

From a velocity point of view, AI accelerates development by automating repetitive test authoring, maintenance, and execution while also orchestrating test runs based on risk, historical flakiness, security exposure, and business priority. This results in faster feedback loops, reduced developer wait-times, and earlier surfacing of both functional and security issues. Coverage also improves meaningfully, as AI can generate tests that target untested paths, simulate complex user or attacker behaviors, and validate security controls that humans may overlook. These capabilities allow teams to validate more functionality with less manual effort. And when combined with predictive analytics, AI increases confidence by focusing validation efforts on the areas with the highest-risk and revealing insights that drive informed quality decisions.

However, achieving these gains requires a “role evolution” of existing teams. Engineers must move from being test executors to becoming quality and security orchestrators or conductors. Instead of manually crafting each test, developers and QA engineers should design intelligent, policy-aware frameworks that can generate, adapt, and monitor thousands of test cases. Their role expands to curating data, refining prompts, managing test agents, and building observability into every testable unit.

For Quality Assurance Engineers (QAE), manual bug hunting and risk resolution give way to investigative collaboration with AI agents, exploring risk maps, defect trends, and intelligent diagnostics. This demands a mindset of trust, continuous tuning, and technical curiosity. As these practices become rooted, the role of QAE increases in strategic importance and becomes a force multiplier. Achieving these outcomes depends not just on what AI can do, but also on how organizations enable their teams to work with it.

### 4.2 Practical considerations: overlaps and potential redundancies

Embedding security at the unit, integration, and system or user interface layers is not redundant, but is optimal for risk because it exposes vulnerabilities before release and reduces the chances of unauthorized access, privilege escalation, lateral movement, and compliance violations. Boehm and Basili (2001) synthesize decades of evidence showing that defect removal cost escalates the later an issue is found, which underwrites the return-on-investment of shift-left security in multi-stage testing pipelines.

To focus efforts where it matters the most, organizations should maintain an application inventory and drive security depth with a formal risk function defined as likelihood multiplied by impact. Felderer and Schieferdecker (2014) formalize this in their risk-based testing taxonomy, which aligns risk assessment and test strategy across all phases and provides a framework for selecting and tailoring techniques depending on system criticality. In practice, critical services (ex. that process critical/PII data) sit high on both likelihood and impact and therefore warrant comprehensive controls such as SAST, DAST, and IAST, while internal applications that handle only public information may be assigned a lighter control baseline. Industrial evidence in Felderer and Ramler (2013) shows that this inventory driven prioritization improves resource allocation and test effectiveness in real organizations and can be introduced stepwise without stopping delivery cadence.

We argue that coverage should be multi-method rather than a single tool because static, dynamic, and interactive techniques cover different classes of vulnerabilities. Large empirical studies on real web systems like Qadir et al. (2025) confirm complementary detection profiles in different types of security tests. This ultimately advocates layered gates rather than one-shot scans. To make this efficient, CI and CD pipelines should enforce policy driven quality gates that block merges or releases when high or critical issues appear and allow progress only after remediation or formally recorded risk acceptance, a practice reflected in systematic reviews of continuous practices and CI trade-offs in the research literature (Shahin et al., 2017).

Overlaps between various security tests (across different layers) may help catch the same class of vulnerabilities, but that may be visible in a different execution context. For example, DAST and Pen Test both simulate external attackers probing for vulnerabilities; however, both have a place in team testing strategies today (Badman and Forrest, 2024). Meanwhile, we also acknowledge that such overlaps can turn into wasteful redundancies (noise). For example, if a vulnerability is accepted in a lower layer, it may resurface in a higher layer, leading to redundant triage and acceptance discussions. To avoid redundant noise when multiple layers indicate the same flaw, findings should be correlated and de-duplicated. Triage policies should be made explicit, a need highlighted by empirical studies of how developers respond to static analysis warnings and why poor signal quality leads to under-use of tools (Johnson et al., 2013).

### 4.3 Practical considerations: challenges

Embedding security controls at every layer of the test pyramid offers earlier vulnerability detection and reduced remediation costs, but this architectural choice introduces challenges that teams must carefully navigate.

Based on our initial assessments, the practical challenges for embedding Security Testing in existing QA workflows manifest themselves in three dimensions. First, from a licensing perspective, commercial SAST and DAST platforms typically charge per developer, per scan, or per application, with enterprise-grade solutions representing substantial annual investments that scale with team size and application portfolio. Open-source alternatives reduce direct licensing costs, but shift the burden on tuning and ongoing maintenance. Next, based on Byrne and Solis (2025), DAST scanners that exercise complete application workflows through instrumented browsers can require between one and three hours of execution time for moderately complex web applications with several hundred routes. This forces teams to extend CI timeout thresholds from the typical 15-min limit to multi hour windows, delaying feedback, and consuming expensive CI runner minutes. Finally, IAST instrumentation, while lighter in comparison to DAST, imposes runtime overhead. Industry benchmarks place this drag around 10% per test execution (OX Security, 2025), which accumulates across integration and contract test suites that may execute hundreds or thousands of scenarios per build. We summarize the practical challenges in Table 1.

**Table 1:** Summary of primary value and key challenge applicable due to introduction of various security tests.

**Traditional testing layer**	**Security control introduced**	**Primary value**	**Key challenge**
Layer-1 (Unit tests)	Static application security testing	Analyzes the source code to identify common security vulnerabilities.	[High Monetary Cost] Tests/Scans at layer-1 run frequently, licensing/run cost may be an issue.
Layer-2 (Component tests)	Security controls validations	Verify that an application's security measures work as intended.	[High Monetary Cost] Some teams may run SAST again (with broader context), resulting in increased licensing/run cost.
Layer-3 (Integration tests)	Interactive application security testing	Provides runtime-verified vulnerability detection with full code-level context.	[Higher Execution Time] Integration may run each beta deployment, IAST may introduce runtime overhead of as much as 10% per execution.
Layer-4 (UI/API Testing)	Dynamic application security testing	A black-box testing that simulates attacks against a running application.	[High Execution Time] May introduce 1+ hours of execution time per run.
Layer-5 (Manual and exploratory testing)	Penetration & Chaos Testing	Penetration testing simulates the behavior of an attacker. While, Chaos Testing intentionally injects failures into a system.	[Significantly higher execution time] most expensive form of security & resiliency testing.

We recommend that teams be pragmatic and exercise the same level of testing as application testing. For instance, in the past application teams may configure software to run Layer-1 and Layer-2 (from Pyramid 1.0), on each code commit/build; however, UI/API testing and Manual tests may be on a need by basis. Similarly, SAST and Security Controls can be frequent, while penetration testing can be periodic. Our motivation is to shift left and ensure the same level of focus on security testing as application testing. We highly recommend that teams identify mechanisms that ensure that they concretely define and follow the repeatability of each layer in the Test Pyramid 2.0.

## 5 Future work

Although we have commenced incrementally adopting our concept of the Test Pyramid 2.0 in our corresponding work, empirical data on productivity, efficiency, and effectiveness are not yet available. As a next step, we will conduct surveys and structured interviews with engineering teams applying this model, gathering both quantitative and qualitative feedback. These results will be used to validate our vision, refine best practices, and identify mitigation measures for any unanticipated challenges.

Another area of focus will be the cost–benefit analysis of the embedded security and AI-driven mechanisms, particularly tools that enabled Interactive Application Security Testing (IAST) and Dynamic Application Security Testing (DAST), which tend to have longer (and more expensive) runs. As covered above, these practices can significantly improve coverage and risk detection, but they can also increase resource consumption, costs, or extend testing timelines if not applied selectively. Measurement of execution times, infrastructure usage, and remediation outcomes will help ensure that these additions deliver net value without making the testing process prohibitively expensive or operationally impractical.

Finally, we will iteratively explore the changes in deployment practices enabled by the adoption of this model. By embedding functional and security testing earlier and automating risk prioritization, we expect that release pipelines will be accelerated while maintaining a high quality bar. We will evaluate how these practices affect the frequency of deployment, rollback rates, and mean time to recovery (MTTR), with the goal of creating a more secure, efficient, and resilient delivery process.

## 6 Conclusion

As modern software systems become more distributed and rapidly evolving, the demands on testing teams have never been higher. This paper presents a concept “The Test Pyramid 2.0,” which integrates Artificial Intelligence and DevSecOps practices to strengthen every layer of the testing stack. We envision that this practical methodology (/road-map) that embeds functional validation, security enforcement, and intelligent automation into a single model, software development can move beyond manual bottlenecks and fragmented efforts to build resilient, scalable, and intelligent quality practices.

Advances in AI enable this transformation by scaling test generation, optimizing execution, improving coverage, and providing contextually appropriate risk detection. DevSecOps ensures that security controls are applied consistently and proactively throughout the delivery process. Together, they shift the paradigm from reactive defect detection to predictive, policy-driven, and risk-focused validation.

However, realizing these benefits is not only a matter of technology. It depends on the evolution of the culture, in which developers, QA engineers, and security specialists evolve to become architects of an intelligent, secure, and resilient quality pipeline. The outcome is a framework that enables organizations to deliver faster without compromising trust.

## References

[B1] Acharya J. Ginde G. (2025). Can we enhance bug report quality using LLMS? An empirical study of LLM-based bug report generation. arXiv preprint arXiv:2504.18804.

[B2] Adu G. (2024). Artificial intelligence in software testing: Test scenario and case generation with an ai model (gpt-3.5-turbo) using prompt engineering, fine-tuning and retrieval augmented generation techniques. Master's thesis, It-Suomen yliopisto.

[B3] Almeida Y. Albuquerque D. Filho E. D. Muniz F. de Farias Santos K. Perkusich M. . (2024). Aicodereview: Advancing code quality with AI-enhanced reviews. SoftwareX 26, 101677. doi: 10.1016/j.softx.2024.101677

[B4] Andrades G. (2025). Agentic Automation in Testing: Smarter Work Flows, Faster Results. ACCELQ. Available online at: https://www.accelq.com/blog/agentic-automation/ (Accessed October 17, 2025).

[B5] Anjali C. Dhas J. P. M. Singh J. A. P. (2023). Automated program and software defect root cause analysis using machine learning techniques. Automatika 64, 878–885. doi: 10.1080/00051144.2023.2225344

[B6] Badman A. Forrest A. (2024). What is Dynamic Application Security Testing (dast)? IBM. Available online at: https://www.ibm.com/think/topics/dynamic-application-security-testing (Accessed October 25, 2025).

[B7] Bagherzadeh M. Kahani N. Briand L. (2022). Reinforcement learning for test case prioritization. IEEE Trans. Softw. Eng. 48, 2836–2856. doi: 10.1109/TSE.2021.3070549

[B8] Baqar M. Khanda R. (2025). Intelligent Computing: Proceedings of the 2025 Computing Conference, Volume 2. Cham: Springer Nature Switzerland.

[B9] Basheer N. Islam S. Alwaheidi M. K. S. Papastergiou S. (2024). Adoption of deep-learning models for managing threat in API calls with transparency obligation practice for overall resilience. Sensors 24:4859. doi: 10.3390/s24154859 39123906 PMC11314962

[B10] Baudry B. Etemadi K. Fang S. Gamage Y. Liu Y. Liu Y. . (2024). Generative ai to generate test data generators. IEEE Softw. 41, 55–64. doi: 10.1109/MS.2024.3418570

[B11] Behjati R. Arisholm E. Bedregal M. M. Tan C. (2019). “Synthetic test data generation using recurrent neural networks: a position paper,” in Proceedings of the 7th International Workshop on Realizing Artificial Intelligence Synergies in Software Engineering, RAISE '19 (IEEE Press), 22–27. doi: 10.1109/RAISE.2019.00012

[B12] Boehm B. Basili V. R. (2001). Software defect reduction top 10 list. Computer 34, 135–137. doi: 10.1109/2.962984

[B13] Byrne J. Solis J. (2025). Tips to Configure Browser-Based Dast Scans. Gitlab. Available online at: https://about.gitlab.com/blog/tips-to-configure-browser-based-dast-scans/ (Accessed October 24, 2025).

[B14] CGFT Engineering (2025). Codebase-Specific RL: Fine-Tuning LLMs for Generating Unit Tests That Boost Coverage. Available online at: https://www.cgft.io/blog/rl-unit-test (Accessed October 15, 2025).

[B15] Chang J. Shi L. Li Z. Zuo X. Hou B. (2025). Security detection algorithm using CNN: anomaly detection for API call sequence. J. Comput. Methods Sci. Eng. 25, 3239–3254. doi: 10.1177/14727978251318813

[B16] Chemnitz L. Reichenbach D. Aldebes H. Naveed M. Narasimhan K. Mezini M. (2023). “Towards code generation from BDD test case specifications: a vision,” in 2023 IEEE/ACM 2nd International Conference on AI Engineering –*Software Engineering for AI (CAIN)*, 139–144. doi: 10.1109/CAIN58948.2023.00031

[B17] Chen Y. Hu Z. Zhi C. Han J. Deng S. Yin J. (2024). “Chatunitest: a framework for LLM-based test generation,” in Companion Proceedings of the 32nd ACM International Conference on the Foundations of Software Engineering, FSE 2024 (New York, NY, USA: Association for Computing Machinery), 572–576. doi: 10.1145/3663529.3663801

[B18] Ćirković S. Mladenović V. Tomić S. Drljača D. Ristić O. (2025). Utilizing fine-tuning of large language models for generating synthetic payloads: enhancing web application cybersecurity through innovative penetration testing techniques. Comput. Mater. Continua 82, 4409–4430. doi: 10.32604/cmc.2025.059696

[B19] Cohn M. (2009). Succeeding with Agile: Software Development Using Scrum. Boston: Addison-Wesley Professional.

[B20] Esposito M. Sarbazvatan S. Tse T. Silva-Atencio G. (2024). The use of artificial intelligence for automatic analysis and reporting of software defects. Front. Artif. Intell. 7:1443956. doi: 10.3389/frai.2024.1443956 39722790 PMC11668792

[B21] Felderer M. Ramler R. (2013). Integrating risk-based testing in industrial test processes. Softw. Qual. J. 22, 543–575. doi: 10.1007/s11219-013-9226-y

[B22] Felderer M. Schieferdecker I. (2014). A taxonomy of risk-based testing. Int. J. Softw. Tools Technol. Transf. 16, 559–568. doi: 10.1007/s10009-014-0332-3

[B23] Fraser G. Arcuri A. (2011). “Evosuite: automatic test suite generation for object-oriented software,” in Proceedings of the 19th ACM SIGSOFT Symposium and the 13th European Conference on Foundations of Software Engineering, ESEC/FSE '11 (New York, NY, USA: Association for Computing Machinery), 416–419. doi: 10.1145/2025113.2025179

[B24] Gamal A. Emad R. Mohamed T. Mohamed O. Hamdy A. Ali S. (2023). “Owl eye: an AI-driven visual testing tool,” in 2023 5th Novel Intelligent and Leading Emerging Sciences Conference (NILES), 312–315. doi: 10.1109/NILES59815.2023.10296575

[B25] Giray G. Bennin K. E. Köksal Ö. Babur Ö. Tekinerdogan B. (2023). On the use of deep learning in software defect prediction. J. Syst. Softw. 195:111537. doi: 10.1016/j.jss.2022.111537

[B26] Hernández-Serrato J. Velasco A. Nifio Y. Linares-Vsquez M. (2020). “Applying machine learning with chaos engineering,” in 2020 IEEE International Symposium on Software Reliability Engineering Workshops (ISSREW), 151–152. doi: 10.1109/ISSREW51248.2020.00057

[B27] Jha P. Scott J. Ganeshna J. S. Singh M. Ganesh V. (2024). “Bertrlfuzzer: a bert and reinforcement learning based fuzzer (student abstract),” in Proceedings of the Thirty-Eighth AAAI Conference on Artificial Intelligence and Thirty-Sixth Conference on Innovative Applications of Artificial Intelligence and Fourteenth Symposium on Educational Advances in Artificial Intelligence, AAAI'24/IAAI'24/EAAI'24 (AAAI Press). doi: 10.1609/aaai.v38i21.30455

[B28] Johnson B. Song Y. Murphy-Hill E. Bowdidge R. (2013). “Why don't software developers use static analysis tools to find bugs?” in 2013 35th International Conference on Software Engineering (ICSE), 672–681. doi: 10.1109/ICSE.2013.6606613

[B29] Klieber W. Flynn L. (2024). Evaluating Static Analysis Alerts With LLMS. SEI Blog. Available online at: https://www.sei.cmu.edu/blog/evaluating-static-analysis-alerts-with-llms/ (Accessed June 19, 2025).

[B30] Komar M. Fedorovych V. Poidych V. Taborovskyi A. (2024). “Intelligent system for visual testing of software products,” in Artificial Intelligence for Sustainable Development 2024.

[B31] Kumar R. (2025). The Agentic AI Testing Revolution: How Intelligent Quality Engineering is Transforming Software Development Forever. Virtuoso QA Blog. Available online at: https://www.virtuosoqa.com/post/agentic-ai-testing-revolution/ (Accessed October 19, 2025).

[B32] Lietz S. (2015). DevSecOps Manifesto. DevSecOps. Available online at: https://www.devsecops.org/ (Accessed May 25, 2025).

[B33] Long L. Wang R. Xiao R. Zhao J. Ding X. Chen G. . (2024). “On LLMs-driven synthetic data generation, curation, and evaluation: a survey,” in Findings of the Association for Computational Linguistics: ACL 2024, eds. L.-W. Ku, A. Martins, and V. Srikumar (Bangkok, Thailand: Association for Computational Linguistics), 11065–11082. doi: 10.18653/v1/2024.findings-acl.658

[B34] Madeyski L. Stradowski S. (2025). Predicting test failures induced by software defects: a lightweight alternative to software defect prediction and its industrial application. J. Syst. Softw. 223:112360. doi: 10.1016/j.jss.2025.112360

[B35] Mathew J. (2025). Achieving Lightning-Fast Parallel Testing with AI. Qyrus. Available online at: https://www.qyrus.com/post/achieving-lightning-fast-parallel-testing-with-ai/ (Accessed October 17, 2025).

[B36] Mehmood M. Amin R. Muslam M. M. A. Xie J. Aldabbas H. (2023). Privilege escalation attack detection and mitigation in cloud using machine learning. IEEE Access 11, 46561–46576. doi: 10.1109/ACCESS.2023.3273895

[B37] Meng R. Mirchev M. Böhme M. Roychoudhury A. (2024). “Large language model guided protocol fuzzing,” in Network and Distributed System Security (NDSS) Symposium 2024. doi: 10.14722/ndss.2024.24556

[B38] Moyer F. (2025). Cucumber Testing: A Key to Generative AI in Test Automation. Kobiton. Available online at: https://kobiton.com/blog/cucumber-testing-a-key-to-generative-ai-in-test-automation/ (Accessed June 28, 2025).

[B39] Nobi M. N. Gupta M. Praharaj L. Abdelsalam M. Krishnan R. Sandhu R. (2022). Machine learning in access control: a taxonomy and survey. arXiv preprint arXiv:2207.01739.

[B40] OX Security (2025). Application Security Testing Guide: Tools & Methods 2025. OX Security. Available online at: https://www.ox.security/blog/application-security-testing/ (Accessed October 22, 2025).

[B41] Pathik B. Sharma M. (2022). Source code change analysis with deep learning based programming model. Autom. Softw. Eng. 29:15. doi: 10.1007/s10515-021-00305-x

[B42] Pyhäjärvi M. (2025). Exploratory Testing With GenAI: How AI Becomes an External Imagination in Software QA. The Qt Company. Available online at: https://www.qt.io/quality-assurance/blog/exploratory-testing-with-genai-how-ai-becomes-an-external-imagination-in-software-qa/ (Accessed July 18, 2025).

[B43] Qadir S. Waheed E. Khanum A. Jehan S. (2025). Comparative evaluation of approaches &tools for effective security testing of web applications. PeerJ Comput. Sci. 11:e2821. doi: 10.7717/peerj-cs.2821 PMC12190248 40567702

[B44] Rashidi H. H. Albahra S. Rubin B. P. Hu B. (2024). A novel and fully automated platform for synthetic tabular data generation and validation. Sci. Rep. 14:23312. doi: 10.1038/s41598-024-73608-0 39375404 PMC11458594

[B45] Rolls C. (2025). AI in Software Development: Productivity Gains, But at What Cost? TTC Global. Available online at: https://ttcglobal.com/what-we-think/blog/ai-in-software-development-productivity-gains-but-at-what-cost/ (Accessed May 26, 2025).

[B46] Romeo F. Arena L. Blefari F. Pironti F. A. Lupinacci M. Furfaro A. (2025). “Arpaccino: an agentic-rag for policy as code compliance,” in New Trends in Database and Information Systems, eds. P. K. Chrysanthis, K. Nørvåg, K. Stefanidis, Z. Zhang, E. Quintarelli, and E. Zumpano (Cham: Springer Nature Switzerland), 467–481.

[B47] Russell R. Kim L. Hamilton L. Lazovich T. Harer J. Ozdemir O. . (2018). “Automated vulnerability detection in source code using deep representation learning,” in 2018 17th IEEE International Conference on Machine Learning and Applications (ICMLA), 757–762. doi: 10.1109/ICMLA.2018.00120

[B48] Saboor Yaraghi A. Holden D. Kahani N. Briand L. (2025). Automated test case repair using language models. IEEE Trans. Softw. Eng. 51, 1104–1133. doi: 10.1109/TSE.2025.3541166

[B49] Santos R. Rizvi S. Cesarone B. Gunn W. McConnell E. (2021). “Reducing software vulnerabilities using machine learning static application security testing,” in 2021 International Conference on Software Security and Assurance (ICSSA), 43–46. doi: 10.1109/ICSSA53632.2021.00016

[B50] Shahin M. Ali Babar M. Zhu L. (2017). Continuous integration, delivery and deployment: a systematic review on approaches, tools, challenges and practices. IEEE Access 5, 3909–3943. doi: 10.1109/ACCESS.2017.2685629

[B51] Shiri Harzevili N. Boaye Belle A. Wang J. Wang S. Jiang Z. M. J. Nagappan N. (2024). A systematic literature review on automated software vulnerability detection using machine learning. ACM Comput. Surv. 57, 1–36. doi: 10.1145/3699711

[B52] Smith H. (2024). AI-Enhanced Test Coverage Analysis and Expansion Through Machine Learning Algorithms. ResearchGate. Available online at: https://www.researchgate.net/publication/386380351_AI-Enhanced_Test_Coverage_Analysis_and_Expansion_through_Machine_Learning_Algorithms (Accessed July 7, 2025).

[B53] Takerngsaksiri W. Charakorn R. Tantithamthavorn C. Li Y.-F. (2025). Pytester: Deep reinforcement learning for text-to-testcase generation. J. Syst. Softw. 224:112381. doi: 10.1016/j.jss.2025.112381

[B54] Terhorst-North D. (2006). Introducing bdd. Dan North & Associates Limited. Available online at: https://dannorth.net/blog/introducing-bdd (Accessed July 11, 2025).

[B55] Varga A. (2025). A Machine Learning-enhanced web-crawler for vulnerability detection: A binary classification approach. PhD thesis, Blekinge Institute of Technology, Faculty of Computing.

[B56] Vocke H. (2018). The Practical Test Pyramid. martinfowler.com. Available online at: https://martinfowler.com/articles/practical-test-pyramid.html#TheTestPyramid (Accessed May 14, 2025).

[B57] Wang C. Zhang W. Su Z. Xu X. Xie X. Zhang X. (2025). “LLMDFA: analyzing dataflow in code with large language models,” in Proceedings of the 38th International Conference on Neural Information Processing Systems, NIPS '24 (Red Hook, NY, USA: Curran Associates Inc.).

[B58] Wang Y. Le H. Gotmare A. D. Bui N. D. Q. Li J. Hoi S. C. H. (2023). “Codet5+: Open code large language models for code understanding and generation,” in Conference on Empirical Methods in Natural Language Processing. doi: 10.18653/v1/2023.emnlp-main.68

[B59] Wen J. Chen Z. Zhu Z. Sarro F. Liu Y. Ping H. . (2025). LLM-based misconfiguration detection for aws serverless computing. ACM Trans. Softw. Eng. Methodol. Just Accepted. doi: 10.1145/3745766

[B60] Yang W. Wang E. Gui Z. Zhou Y. Wang B. Xie W. (2025). An mllm-assisted web crawler approach for web application fuzzing. Appl. Sci. 15:962. doi: 10.3390/app15020962

[B61] Zhang J. Hu X. Xia X. Cheung S.-C. Li S. (2025). Automated unit test generation via chain of thought prompt and reinforcement learning from coverage feedback. ACM Trans. Softw. Eng. Methodol. Just Accepted. doi: 10.1145/3745765

